# Meta-analytic rain cloud plots: Improving evidence communication through data visualization design principles

**DOI:** 10.1017/rsm.2025.4

**Published:** 2025-03-10

**Authors:** Kaitlyn G. Fitzgerald, David Khella, Avery Charles, Elizabeth Tipton

**Affiliations:** 1 Azusa Pacific University, Azusa, CA, USA; 2 Villanova University, Villanova, PA, 19085, USA; 3 Northwestern University, Evanston, IL, 60208, USA

**Keywords:** data visualization, forest plot, MARC plot, meta-analysis, statistical cognition

## Abstract

Results of meta-analyses are of interest not only to researchers but often to policy-makers and other decision-makers (e.g., in education and medicine), and visualizations play an important role in communicating data and statistical evidence to the broader public. Therefore, the potential audience of meta-analytic visualizations is broad. However, the most common meta-analytic visualization – the forest plot – uses non-optimal design principles that do not align with data visualization best practices and relies on statistical knowledge and conventions not likely to be familiar to a broad audience. Previously, the Meta-Analytic Rain Cloud (MARC) plot has been shown to be an effective alternative to a forest plot when communicating the results of a small meta-analysis to education practitioners. However, the original MARC plot design was not well-suited for meta-analyses with large numbers of effect sizes as is common across the social sciences. This paper presents an extension of the MARC plot, intended for effective communication of moderate to large meta-analyses (*k* = 10, 20, 50, 100 studies). We discuss the design principles of the MARC plot, grounded in the data visualization and cognitive science literature. We then present the methods and results of a randomized survey experiment to evaluate the revised MARC plot in comparison to the original MARC plot, the forest plot, and a bar plot. We find that the revised MARC plot is more effective for communicating moderate to large meta-analyses to non-research audiences, offering a 0.30, 0.34, and 1.07 standard deviation improvement in chart users’ scores compared to the original MARC plot, forest plot, and bar plot, respectively.

## Highlights

**What’s already known**

- Forest plots are the most widely used meta-analytic visualization, yet the forest plot design does not align with best practices established in the data visualization literature.
- The Meta-Analytic Rain Cloud (MARC) plot is an effective alternative to a forest plot when communicating the results of small meta-analyses (*k* = 5) to education practitioners.

**What’s new**

- This paper presents an extension of the MARC plot, intended for effective communication of moderate to large meta-analyses (*k* = 10, 20, 50, 100).
- The MARC plot design incorporates best practices from data visualization and cognitive science for evidence communication, with the course of expertise and non-researcher audiences in mind.
- We evaluate the MARC plot in a randomized survey experiment and find it continues to be a more effective alternative to a forest plot for moderate and large meta-analyses.

**Potential impact for *research synthesis methods* readers**

- The MARC plot can be used as an effective visualization for communicating the results of a meta-analysis to a general audience.
- Practitioners (e.g., in education or medicine) consult meta-analyses in their decision-making, so the potential audience of meta-analytic visualizations is broad.

The curse of expertise complicates the communication of research findings to non-researchers; this paper can help the reader overcome this difficulty by thinking more critically about evidence communication and data visualization best practices.

## 1 Introduction

In the social sciences, meta-analyses often include as many as 40 or even 100 studies,^1^ ^,^ ^2^ and these studies can include even more effect sizes (upwards of 400). As the number of studies and effect sizes have increased, the role of meta-analysis has shifted from simply providing an estimate of the average effect size, to quantifying the degree of variation in effects and explaining this variation via moderators.^3^ In fields like education and psychology, the results of these meta-analyses are often of interest to not only scientists and researchers but also to decision-makers in schools and communities.

Data visualizations provide an important tool for providing the results of analyses to the broader public. Such visualizations are increasingly present in newspapers – including the New York Times, Wall Street Journal, and websites like Five-Thirty-Eight. These visualizations have become an important part of how the public interprets research findings, including results of public polling, experimental findings, and presidential election predictions. Over time, these visualizations have evolved based on feedback from readers and the public. An example that many point to is the way in which predictions were displayed in the 2016 U.S. presidential election between Hilary Clinton and Donald Trump; these visualizations were unfortunately misinterpreted, often leading readers to believe that Clinton would nearly certainly win the election, when in fact, a Trump win was far from unlikely.

In meta-analysis, visualizations of data – including funnel plots, forest plots, distribution plots, and bubble plots – have long been common,^4^ ^,^ ^5^ and the ability to produce these plots is included in nearly every major and minor software package used by analysts.^4^ Of these, the forest plot is the most reported.^4^ ^,^ ^5^ Forest plots illustrate meta-analytic data in highly standardized ways. To do so, estimated effect sizes are indicated with boxes (whose size is typically proportional to weight), and lines extend on either side to indicate 95% confidence intervals. At the bottom, a pooled effect size and its 95% confidence interval are indicated using a larger diamond.^6^ ^,^ ^7^ Their widespread use is not surprising given they are recommended in the PRISMA Statement^8^ ^,^ ^9^; this recommendation has led researchers to report forest plots even when there is no data to include.^5^

The widespread use of forest plots is based upon their ability to convey findings—particularly related to heterogeneity—to others in the meta-analysis community. As Schriger et al.^5^ note,

“A standardized format for forest plots no doubt helps readers because *repeated exposure to a familiar format* decreases the time and effort required to become oriented to the graphic and likely facilitates their interpretation.” (Schriger et al.^5^; italics added)

However, meta-analysts are not the only community turning to meta-analyses for information. These broader audiences—including scientists and researchers producing primary analyses, decision-makers making policies and selecting programs (e.g., in schools, and hospitals), and the general public—have far less experience with forest plots and meta-analyses. On its own, this would not be problematic, but as Schriger et al.^5^ note, many of the features and standards for forest plots are not based on best practices or theory in the broader field of data visualization, and theory from these fields suggests, in fact, that “current practice is suboptimal.” This recognition can be seen, too, in later work by Nakagawa et al.^10^—developing an orchard plot extension to the forest plot—and by Schild and Voracek^11^—developing the rainforest plot. Interestingly, however, Schild and Voracek^11^ is to date the only such paper on forest plots that includes both theoretical development and empirical testing of the forest plot and possible refinements.

In this paper, we develop and test an alternative to the forest plot that is meant to convey effect sizes and meta-analysis results from large reviews to the broader public. This plot called the Meta-Analytic Rain Cloud (MARC) Plot, was first introduced by Fitzgerald and Tipton^12^ for use in research clearinghouses and with small meta-analyses including at most five studies. The plot was developed based on state-of-the-art theory and developments in data visualization and was tested using a sample of both researchers (familiar with forest plots) and non-researchers. The findings of that study indicated that the MARC Plot outperformed both the forest plot and the rainforest plot. However, reviewers and others remained concerned that while the MARC Plot could well convey the findings of these small reviews, such a plot would not continue to be useful and successful with the larger meta-analyses found in the social sciences. In this paper, we therefore modify and test the MARC Plot with meta-analyses including larger numbers of studies (up to 100). We then test this with a general audience of college-educated adults using a survey experiment. Meta-analytic visualizations typically have textual information that accompanies each effect size (e.g., Study ID), and this can quickly clutter the visual as the number of effect sizes increases. Therefore, we made all visualizations in the experiment interactive in order to maintain readability. The findings of this large-sample version of the interactive MARC Plot are consistent with earlier findings (of the small-sample static version), indicating that the type of data visualization that is appropriate for a broad audience differs from that useful for the meta-analytic community alone.

This paper is structured as follows: In Section 2, we provide a brief review of problems with forest plots based on the data visualization and cognitive psychology literature. In Section 3, we review the development of the original small-sample MARC Plot and two possible extensions that could work in larger samples. In Section 4, we provide an overview of the methods through which we tested the effectiveness of these plots, including how we recruited our sample, our factorial experimental design, and the development of measures of performance. In Section 5, we provide results from the survey experiment. We conclude the paper in Section 6, including a discussion of implications for the use of the MARC Plot in the broader field.

## 2 Why a new meta-analytic visualization is warranted

A crucial lesson offered by the cognitive science and data visualization literature is that as researchers we must beware of the *curse of expertise.* This is a well-documented psychological phenomenon wherein people who hold some form of expert knowledge severely overestimate the extent to which other people hold that same knowledge and will interpret information similarly.^13^ Additionally, the communication of meta-analytic evidence involves the communication of statistical ideas such as effect sizes and their uncertainty, yet the statistical cognition literature has documented many widespread and persistent cognitive pitfalls that often hinder appropriate statistical reasoning.^14^ ^–^ ^17^ This combination—the curse of expertise and widespread difficulties in statistical reasoning—means the communication of meta-analytic evidence to non-researchers is an area ripe for careful development of best practices.

**Figure 1:**
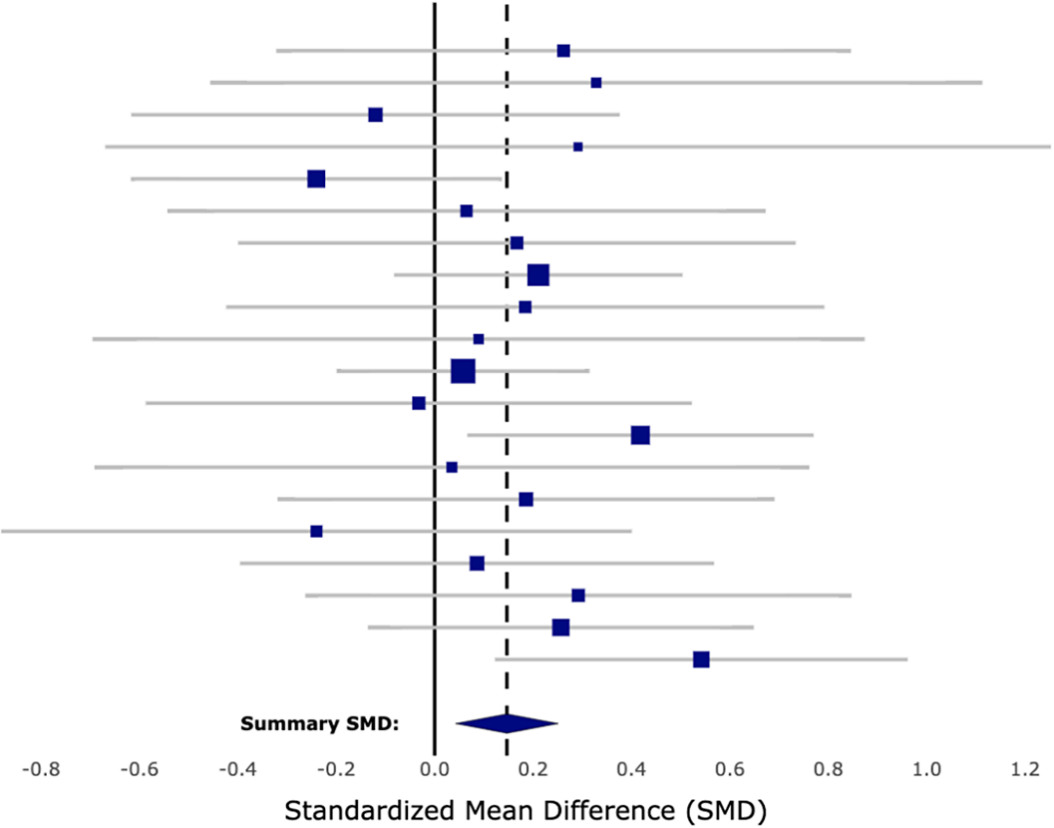
Forest plot of k = 20 studies.

Forest plots (e.g., Figure 1) have been a long-standing visualization of choice for researchers communicating meta-analytic evidence, so we begin here by discussing the anatomy of forest plots in light of what the data visualization and cognitive science literature suggests may complicate users’ interpretations of them.

In data visualization design, it is customary to carefully consider the “encodings” that are used to map information from a dataset to aesthetic elements of the visualization. The goal of an effective visualization is to choose encodings that make key information salient to the audience and guide them toward the desired interpretation.^18^ Depending on the “story” a chart creator is trying to communicate, some design choices and encodings will be more effective than others. In a meta-analytic visualization, the implicit “story” being told is the overall summary of the evidence as well as how that summary came to be as an aggregate of individual effect sizes. In order to tell this story, the key information in a meta-analytic dataset that must be encoded in a visualization is effect size magnitude (e.g., standardized mean difference or odds ratio) and effect size uncertainty (e.g., standard error, confidence interval, and/or meta-analytic weight).

In a forest plot, individual effect sizes are encoded as squares, with the effect size magnitude mapped to the *x*-axis position of the center of the square and its meta-analytic weight mapped to the area of the square. These encoding choices result in two useful visual properties of a forest plot: (1) larger positive effect sizes appear farther to the right (making effect size magnitude salient) and (2) more precise effect sizes are represented by larger squares (making effect size precision salient). However, these visual messages get muddled by the fact that the precision of effect sizes is *also* displayed with error bars. In a forest plot, confidence intervals are encoded as bars extending from the lower bound to the upper bound, again utilizing the *x*-axis position. This double-use of the *x*-axis works counterproductive to both of the useful properties listed above. First, the bars that extend farthest to the right do not meaningfully correspond to the effect sizes with the largest magnitude. But more detrimentally, the longest bars (widest intervals) correspond to the *least precise* effect sizes, thus bringing them undue visual attention. Fitzgerald and Tipton^12^ found that when education decision-makers were asked “which study was given the most weight in determining the summary SMD” or “which study’s findings do you trust the most,” less than 60% of participants were able to answer correctly when viewing a forest plot. The most common wrong answer to both was choosing the effect size with the *widest* confidence interval as the one that got the most weight or that they trusted the most. In a meta-analysis—where inverse-variance weighting is essential to optimal synthesis—this is exactly the opposite of the statistical reasoning we want our audience to employ. In other words, non-researchers do not appear to possess the same intuition that square size corresponds to weight nor an understanding of how that should factor into evidence aggregation. Indeed, the curse of expertise appears to plague researchers’ communication and non-researchers’ interpretation of forest plots. The encoding of the summary effect’s precision as the width of the diamond is perhaps even more opaque for those unfamiliar with meta-analysis.

An additional feature of the forest plot that is non-optimal according to the data visualization literature is the fact that it does not meaningfully utilize the *y*-axis position. Cognitive scientists have created a hierarchy of visual encodings in terms of how quickly and accurately the human brain’s visual system is able to extract information. Position encodings (i.e., *x*-axis or *y*-axis position) sit at the top of this hierarchy, with accurate data extraction occurring faster than an eyeblink.^18^ ^,^ ^19^ In a forest plot, the *y*-axis is typically used to sort studies arbitrarily by last name or year of publication, but it is not leveraged in such a way that helps users reason about the effect sizes or their aggregation.

## 3 Design of the MARC plot and motivation for the current experiment

**Figure 2:**
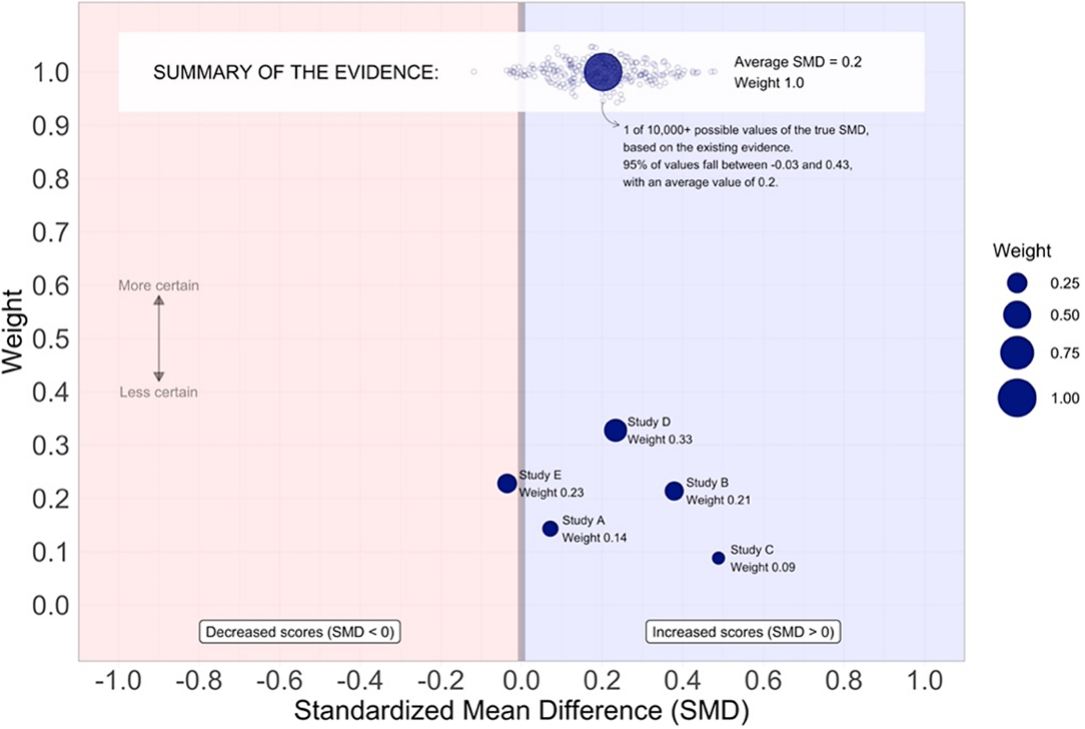
Meta-analytic rain cloud (MARC) plot—original design.

Fitzgerald and Tipton^12^ proposed the Meta-Analytic Rain Cloud (MARC) plot as an alternative way of visualizing meta-analytic data from research clearinghouses. Figure 2 displays the original MARC plot design. Here we will highlight key design features. The MARC plot encodes effect sizes as dots, with the effect size magnitude mapped to the *x*-axis position (similar to a forest plot) and the meta-analytic weight mapped to the *y*-axis position (dissimilar to a forest plot). The size of the dot also encodes the meta-analytic weight. This gives the desirable visual property that more precise studies appear both higher up on the *y*-axis and as larger dots, thus visually (de-)emphasizing effect sizes in a manner consistent with normative meta-analytic reasoning. We chose to not include confidence intervals on the individual effect sizes both the avoid the counter-productive visual encodings discussed for the forest plot but also to minimize potential vote-counting behavior that may be more common among non-researcher audiences. Additionally, we added annotations and informal legends to aid in the interpretation of the evidence; the “More certain ← →Less certain” arrows make explicit the connection between certainty and weight, and the red/blue shading and the “Decreased/Increased scores” labels guide towards appropriate interpretations of positive and negative standardize mean differences. Finally, we placed the summary effect at the top and displayed its confidence interval as a “beeswarm,” following evidence that frequency displays rather than aggregate probabilities and continuous displays rather than dichotomous ones (e.g., error bars) improve reasoning about uncertainty.^18^ ^,^ ^20^ ^–^ ^22^

Fitzgerald and Tipton^12^ evaluated the MARC plot in comparison with a forest plot, a rainforest plot,^11^ and a bar plot, which is commonly used by the What Works Clearinghouse for displaying meta-analytic data. We found that the MARC plot did facilitate improved interpretation of evidence from small meta-analyses among a sample of education decision-makers and researchers.

**Figure 3:**
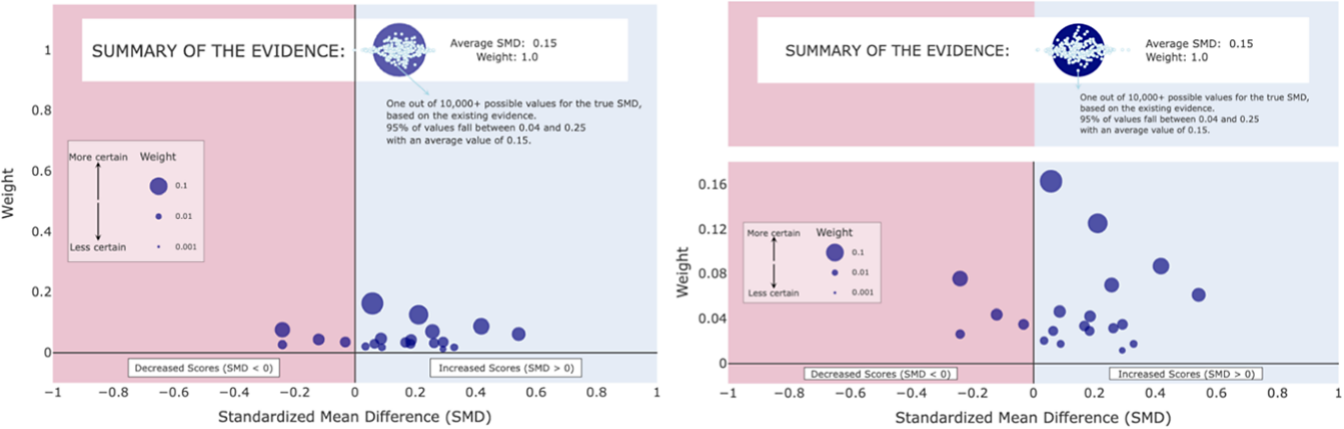
MARCv1 (left) and MARCv2 (right) for k = 20 studies.

While this prior work provided promising evidence for the MARC plot, it only investigated the case when there were *k* = 5 studies in the meta-analysis. Important questions remain as to whether the efficacy of the MARC plot persists in visualizing evidence from larger meta-analyses, as are more common in the social sciences. Of particular concern is whether or not the *y*-axis positional encoding of weight will remain effective; as the number of studies in a meta-analysis increases, the relative weight of each effect size will inherently shrink. This will result in the effect size dots appearing to “pool” at the bottom of the visualization, near the *x*-axis (see Figure 3a). On the one hand, this may be a desirable result in that it de-emphasizes individual studies and potentially places more emphasis on the meta-analytic summary. On the other hand, this may result in a cluttered visualization that loses the visual advantages of the original MARC plot.

We designed an adjusted MARC plot (Figure 3b) to ameliorate this concern for displaying evidence from large meta-analyses. Here, the summary effect is put on a separate plane that is no longer on the same *y*-axis scale as the individual studies. While this potentially obscures the summary effect’s aggregate nature with a combined weight of *y* = 1.0, the change allows the lower pane to still show meaningful differences in relative weights among the individual effect sizes rather than having them pooled at the bottom.

In the sections that follow, we detail the statistical cognition experiment we conducted to evaluate the efficacy of these two versions of the MARC plot compared with a forest plot and a bar plot when displaying moderate-to-large meta-analyses (*k* = 10, 20, 50, 100). The experiment also generates new evidence regarding meta-analytic reasoning among a more general audience.

## 4 Methods

This study was conducted online via Qualtrics. Our research questions, experimental design, questionnaire, power analysis, data collection plan, and analysis plan were pre-registered with the Open Science Foundation (OSF) prior to data collection. The full pre-registration along with additional online supplementary material can be found in the following OSF repository: https://osf.io/49ck7/.

### 4.1 Research questions

In this study, we ask four questions:

1. Are users able to accurately interpret meta-analytic visualizations for meta-analyses with a moderate to large number of studies?
2. Which type of visualization leads to the most accurate understanding among users?
3. Do the visualizations perform consistently for all levels of *k* (no. of studies)?
4. Does the length of time spent answering questions vary by visualization type? Does this relationship depend on *k*?

In particular, we are interested in which of the two MARC plots is best, and whether or not the advantage of the MARC plot over the bar plot and forest plot (as seen in Fitzgerald and Tipton^12^) persists in the context of meta-analyses with larger numbers of studies. While bar plots are not as ubiquitous as forest plots for displaying meta-analytic data, we include them in this study as they are used by the What Works Clearinghouse (a primary clearinghouse for U.S. education research) and are common for visualizing effect sizes more broadly.^20^ Because of their simplicity, there is a temptation to assume they might be well-suited for communicating with non-researcher audiences. However, as discussed in Fitzgerald and Tipton,^12^ they suffer from some of the same design flaws as forest plots (e.g., *y*-axis not utilized, visual attention misaligned with meta-analytic weight). We hypothesize that users may have difficulty interpreting and make errors in meta-analytic reasoning with bar plots and forest plots, but that the MARC plots, especially the adjusted MARC plots, will lead to the most accurate understanding. We anticipate that visualizations may become more difficult to interpret as *k* increases and that this may be reflected in lower scores and longer viewing time. However, we anticipate this relationship may not be the same for all visualizations—that is, we expect an interaction effect between visualization type and number of studies may be present. We hypothesize that viewing time will vary across visualizations.

### 4.2 Experimental design

We used a 4 × 4 factorial design with the following factors and levels:

- Factor A: Visualization Type (MARCv1, MARCv2, BP, FP).
- Factor B: *k*, the number of studies in the meta-analysis (10, 20, 50, 100).

Crossing these two factors gives 16 treatment combinations. However, since it would be unfeasible to have every participant answer questions about all 16 visualizations, we confounded the treatment combinations into 4 blocks. Each participant therefore viewed 4 visualizations. With a sample size of 160, 10 replicates of the full 16-run design were possible. We used three different confounding patterns in order for interaction terms of interest to be estimable in at least some of the replicates. We also chose the confounding patterns such that each visualization type was represented in every block (i.e., each person viewed one MARCv1, one MARCv2, one bar plot, and one forest plot, each with potentially varying *k*). This ensures that Factor A (visualization type) was estimable within people, making for a more powerful design. Details of the confounding patterns can be found in Online Appendix A in the OSF repository.

To implement the experimental design, the Qualtrics survey was designed to have 12 survey blocks (4 experimental blocks * 3 confounding patterns). We used the “Randomizer” feature in Qualtrics to randomly assign participants into one of the 12 survey blocks and the “Evenly Present Elements” option to ensure roughly equal sample sizes in each block.^23^

### 4.3 Questionnaire

The same questionnaire as Fitzgerald and Tipton^12^ was used, with the addition of one question (Q8) for exploratory purposes. Participants were asked to answer the questions in Table 1 for each of the four visualizations they viewed. The intention of these questions is not to capture the complexity of decision-makers reasoning about the evidence but rather to simply assess whether participants are accurately able to extract information necessary for making a normative statistical judgment about the evidence. All of these questions have objectively correct answers determined by statistical and meta-analytic norms; therefore, participant responses to each question were scored as 0 or 1 if they answered incorrectly or correctly, respectively. As delineated in the pre-registration, Question 8 was included for exploratory purposes only and was therefore excluded from the score calculation. The participants’ score was calculated out of 7 points as in Fitzgerald & Tipton.^12^ With the exceptions of Q5 and Q8, all of these questions were multiple-choice. Q5 had a slider (ranging from −0.5 to 0.5), and Q8 asked users to manually input in a lower and upper bound for their range of values. A full version of the questionnaire including answer choices and Qualtrics survey logic can be found in the OSF repository.

**Table 1:** Survey questionnaire

ID	Question text
Q1	Which study’s findings do you ***trust* *the most***?
Q2	Which study was given the ***most*** weight in determining the summary SMD?
Q3	Which study’s results are the ***least*** certain?
Q4	Which study found that the new curriculum improved scores by the ***greatest*** amount?
Q5	On average, how much did the new curriculum increase or decrease student scores?
Q6	Which of the following provides the ***best (that is most certain)*** estimate of the true SMD?
Q7	Is there sufficient evidence to conclude that the new curriculum improves scores (that the true SMD > 0)?
Q8	Based on the summary evidence, please report a plausible range of values the true SMD might reasonably be between.

*Note:* Text emphasis appears exactly as it did to survey participants.

### 4.4 Participants

We recruited participants via the online platform Prolific. Prolific is a platform similar to Amazon’s Mechanical Turk that connects researchers with survey participants and includes several quality control features to ensure quality data. Participants had to have an established Prolific profile and meet the following screening criteria in order to participate in the study:

1. Be located in the United States
2. Speak English as a first language
3. Hold a Bachelor’s degree or higher

The educational requirement is intended to reflect an audience that has a similar level of education to education decision-makers (e.g., principals, district superintendents)—those who are college-educated but may not necessarily have research or statistical training. The use of crowd workers is common practice in data visualization and human-computer interaction studies, however, it should be noted that the fact our sample is not comprised specifically of education decision-makers is a limitation. In addition to the screening requirements, we excluded participants if they failed an attention check built into the survey (*n* = 17) or if they finished in under 3 min (*n* = 0). Demographic characteristics of the included 160 participants can be seen in Table 2. The sample size was determined by a pre-registered power analysis. Details can be found in Online Appendix B in the OSF repository.

**Table 2:** Participant demographics (n = 160)

	*n*	Proportion
Gender		
Female	57	0.356
Male	101	0.631
Non-binary/third gender	1	0.006
Prefer not to say	1	0.006
Age		
18–24	9	0.056
25–34	47	0.294
35–44	44	0.275
45–54	31	0.194
55–64	21	0.131
65 or older	8	0.050

### 4.5 Generating meta-analytic data

We generated four meta-analytic datasets associated with the four different values of *k* for Factor B in the experimental design. To simulate data reasonable for the education research context, we assumed each study was a cluster-randomized trial (CRT), and the effect size parameter of interest within a study was a standardized mean difference,

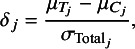

where 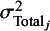 is the total variance in study *j* that incorporates the within- and between-cluster variance. Across the four levels of *k* (10, 20, 50, 100), we generated effect sizes and sample sizes for a total of 180 studies. We assume one effect size per study and the effect size estimates 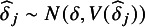 , where 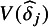 is computed as a function of 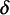 , the intraclass correlation, 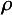 , and the total, within-cluster, and within-treatment group sample sizes, according to formula (16) in Hedges and Hedberg (2016). Note this follows the standard assumption in meta-analytic models that each effect size provides an unbiased estimate of the treatment effect. We set 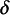 = 0.15, which represents a small effect in the education context, and assumes an intraclass correlation of 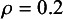 .^24^ Using sample size data from the What Works Clearinghouse as a guide for our data generation parameters,^25^ we generated sample sizes (number of clusters and number of students within clusters) for the 180 studies using the mvlognormal() from the MethylCapSig R package.^26^ We assumed equal sample sizes in treatment and control groups. We then used the simulated sample sizes to compute the standard errors for each 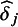 , which in turn determined the corresponding meta-analytic weights 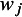 . For simplicity of data generation, we assumed a fixed-effects meta-analytic model so that 
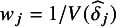
 . The R code for this data generation as well as the generated data can be found in the OSF repository. The meta-analytic summary data for the four generated datasets can be seen in Table 3.

**Table 3:** Meta-analytic summaries of data for 4 experimental levels

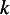	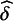	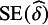	Lower bound 95% CI	Upper bound 95% CI
10	0.147	0.070	0.009	0.285
20	0.147	0.053	0.043	0.251
50	0.145	0.029	0.088	0.203
100	0.156	0.022	0.113	0.200

### 4.6 Creating the visualizations

**Figure 4:**
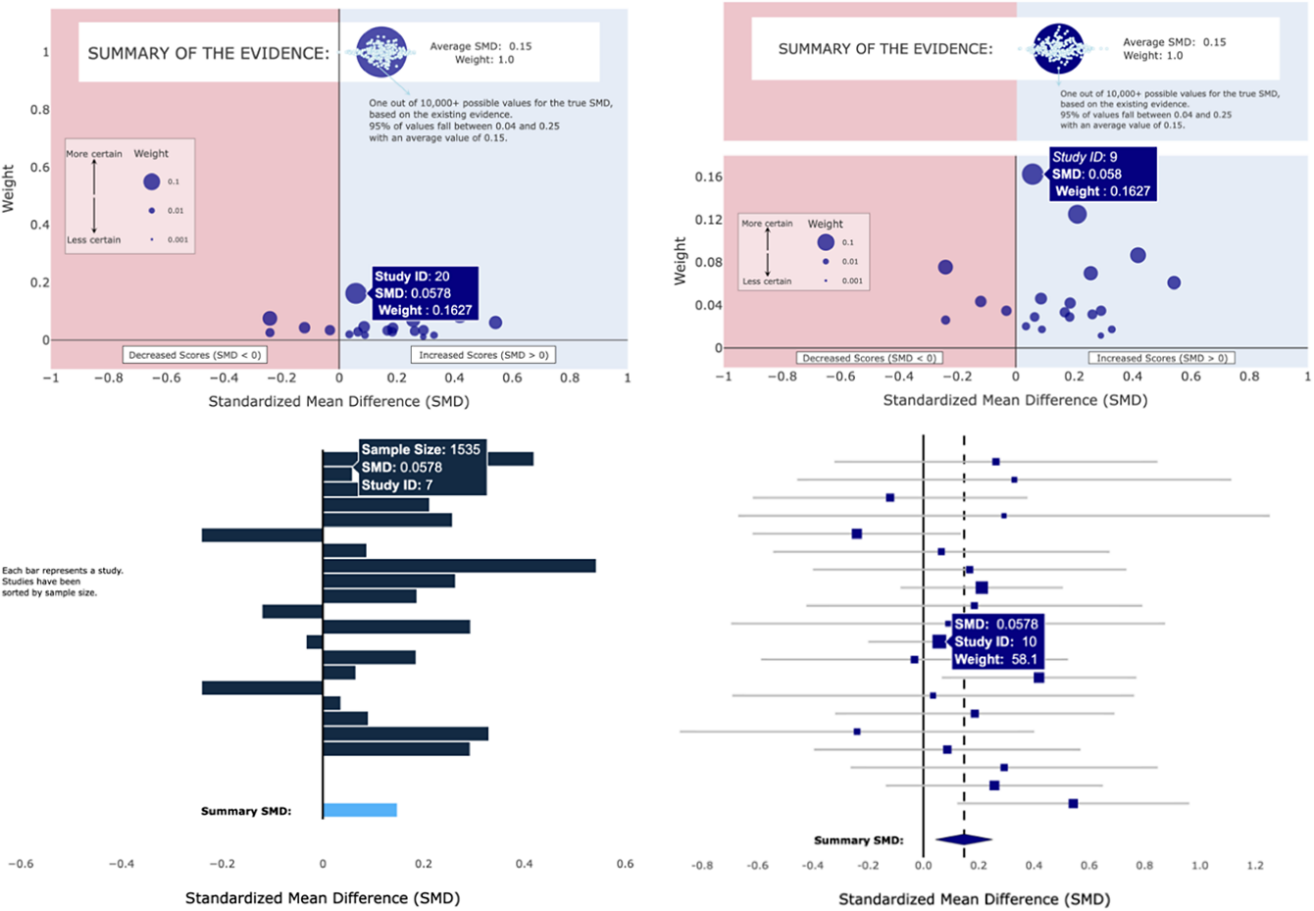
Four visualizations of the same k = 20 studies. MARC plot version 1 (top left), MARC plot version 2 (top right), Bar plot (bottom left), Forest plot (bottom right).

We displayed each of the 4 meta-analytic datasets (*k* = 10, 20, 50, 100) as each visualization type (MARCv1, MARCv2, BP, FP), for a total of 16 experimental visualizations. All visualizations were created in R version 4.3.0. We made each visualization interactive with key study information visible via mouse hovering instead of directly on the visualization, in order to avoid clutter as the number of studies in the visualizations increased. For all visualization types, hovering over a particular study would display its Study ID and SMD estimate as well as the measure of uncertainty embedded in the original static design; bar plots display sample size (# of students), forest plots display raw weights, and MARC plots display relative weight. Bar plots and forest plots were created via the ggplot2 R package and converted to interactive displays via the ggplotly() wrapper function from the plotly R package. Some of the features of the MARC plot are not supported by the plotly() function, so the MARC plots were built using plot_ly() directly instead of as a wrapper around ggplot(). We utilized Plotly Chart Studio to publish the interactive visualizations and generated iframe code to embed the visualizations in our Qualtrics survey. All 16 interactive visualizations as well as the R code used to create them can be found in the OSF repository. Static versions of each visualization type for *k* = 20 can be seen below in Figure 4, with an example study’s hover information visible. Note that the same effect size is shown in each of the four visualizations, but the study ID was intentionally randomized for the purposes of the experiment since participants would be answering the same set of questions four times.

### 4.7 Analysis plan

The analysis plan for each of our four primary research questions can be found in the Appendix. All analyses were pre-registered with the Open Science Foundation—including multiple comparison procedures chosen to control the Type I error rate at the level 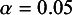 for each analysis. The survey data and R code for all analyses can be found in the OSF repository.

## 5 Results

***Research Question 1:** Are users able to accurately interpret meta-analytic visualizations for meta-analyses with a moderate to large number of studies?*

**Figure 5:**
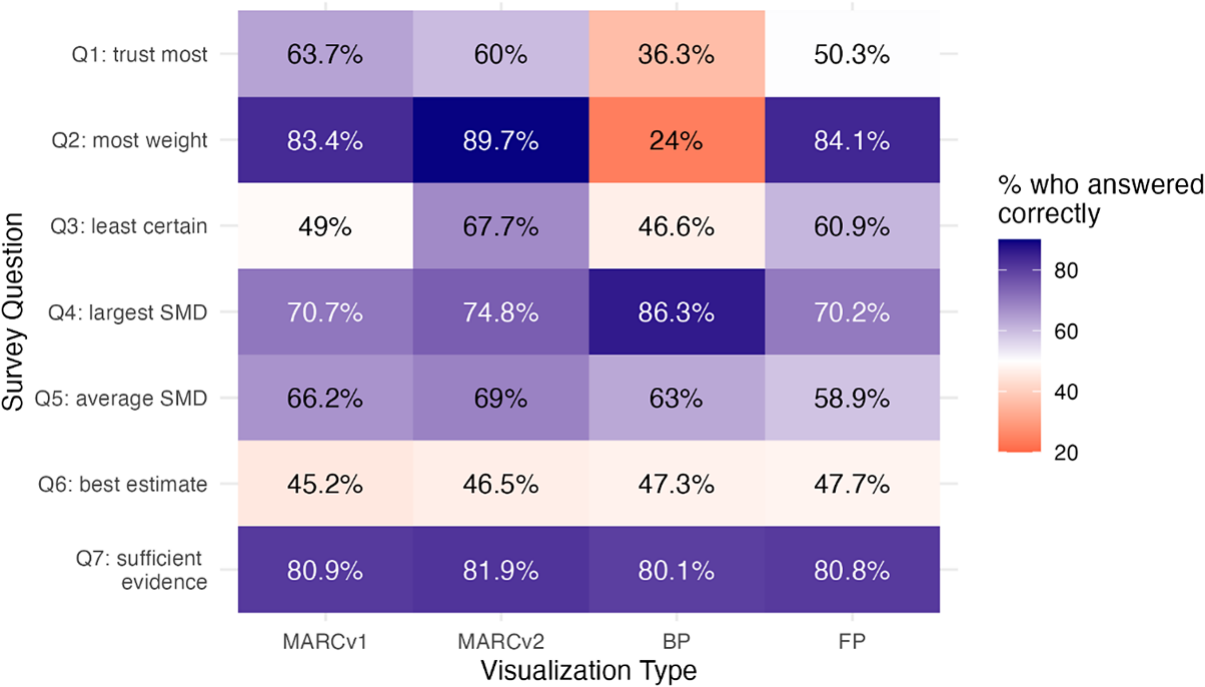
Percentage of participants who answered each question correctly for each visualization type.

Figure 5 shows the proportion of participants who answered each question (rows) correctly for each visualization type (columns). Darker blue colors indicate that participants scored well on that question-visualization combination, whereas darker red colors indicate participants performed poorly; white represents the center of the scale where 50% of participants answered a question correctly for a given visualization type. It is instructive to consider how the results for Q2 (*Which study was given the most weight in determining the summary SMD?)* differ across the four visualizations. Notably, more than 8 in 10 were able to answer this correctly on both MARC plots and the forest plot, whereas less than 1 in 4 were able to do so correctly on the bar plot. The bar plot suffers from two particular challenges, both related to how it provides information about meta-analytic weight *only* via sample size labels for the number of students in the study. The first is that because the data come from cluster-randomized trials - where standard errors are driven more by the number of *clusters* than the number of *students -* student sample size can be a poor proxy for meta-analytic weight. This is not simply a hypothetical problem; indeed, for some of the naturally occurring meta-analytic datasets generated according to the models detailed in Section 4, the study with the largest number of students was *not* the study with the largest meta-analytic weight. Among participants who were shown this dataset via a bar plot, *all answered Q2 incorrectly.* It is worth noting that the forest plot performs better in Q2 in this experiment compared to Fitzgerald & Tipton,^12^ where only 57% answered it correctly when viewing a static forest plot of 5 studies. While we are cautious about drawing direct comparisons, we hypothesize this may be in part because the interactive visualization includes the hover text with study weight *in addition to* the visual encoding of square size, whereas the static visualization has only square size.

From a purely statistical standpoint, Q1 *(Which study’s findings do you trust the most?)* is an identical question to Q2. The fact that participants do relatively worse in Q1 as compared to Q2 for the MARC and forest plots suggests that general audiences may not hold the same intuition or norms that treat studies with more statistical precision as more trustworthy. This gap seems to be mitigated slightly for the MARC plots, perhaps due to the informal legend that indicates that studies higher up on the *y*-axis are more *certain.* More formal study should be conducted to investigate non-researchers notions of (statistical) certainty as it relates to the trustworthiness of evidence.

MARCv1 and MARCv2 perform comparably to one another with Q3 as a notable exception; the “pooling” of effect sizes at the bottom of MARCv1 makes it very difficult to identify studies with low weight, particularly as *k* increases, whereas the adjusted *y*-axis scaling in MARCv2 increases the visual spacing and reduces clutter.

All four visualizations facilitate the extraction of the study with the largest SMD relatively well (Q4), as all four visualizations have this encoded as the *x*-axis position. The higher performance for the bar plot relative to the others on this question is likely because a bar plot has this information also encoded as the *length* of the bar, contributing to the amount of visual attention the largest SMD receives. In the results not shown here, we note that the forest plot did relatively worse on this question as *k* increased, which did not happen for the other visualizations. This is likely because while SMD is encoded as an *x*-axis position, so is the end of the confidence interval bars, so the increased visual clutter with increased studies may make it more difficult to ignore the confidence interval bars and focus on only the squares to discern which one is farthest to the right.

All four visualizations perform poorly on Q6, which asks them to ascertain that the summary effect is the best (that is most certain) estimate of the true SMD, over and above any individual study. More work should be done to investigate whether this is simply due to a misunderstanding of the question, a lack of meta-analytic intuition about the usefulness of the summary effect, or some other reason.

All four visualizations perform well on Q7, which asks whether there is sufficient evidence to conclude the curriculum improves student scores. This is perhaps unsurprising since all generated meta-analytic datasets used in this experiment had statistically significant summary effects. Prior work indicated that the statistical significance of the summary effect was not a significant source of variation in respondents’ interpretation of the evidence, so we chose not to manipulate it as a factor in the present experiment but rather let the standard errors occur naturally according to our chosen simulation parameters. However, future work should investigate how chart users respond to notions of “sufficient evidence” when true heterogeneity is present.

***Research Question 2:** Which type of visualization leads to the most accurate understanding among users?*

**Figure 6:**
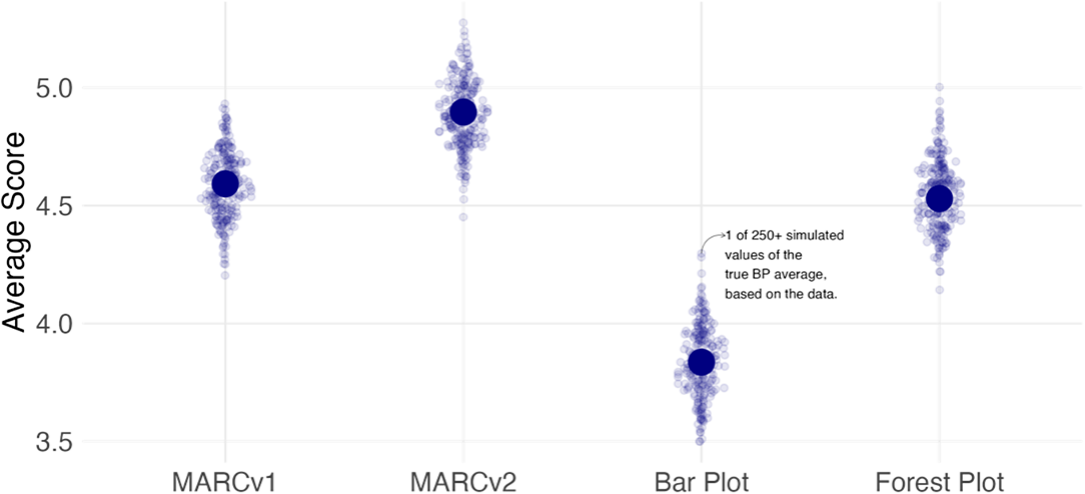
Average participant scores by visualization type.

We find that overall MARCv2 performs best, as shown in Figure 6. Table 4 provides results for Tukey’s pairwise comparisons of the difference in mean scores for each of the 6 visualization pairs. The estimated MSE was roughly 1 for this data, so the raw difference estimates are very similar to the standardized Cohen’s *d* estimates and can be interpreted as such. That is, MARCv2 offered approximately a 1 standard deviation improvement over the bar plot, a 0.37 standard deviation improvement over the forest plot, and a 0.3 standard deviation improvement over MARCv1, all of which were statistically significant. Interestingly, MARCv1 did not perform significantly better than the forest plot, suggesting that the original MARC plot design does not perform as well with large *k* when small relative weights result in the “pooling” of effects at the bottom of the visualization. Therefore, it seems the re-scaling of the *y*-axis as in MARCv2 is important to maintain the usefulness of the *y*-axis encoding and help users discern meta-analytic weight when there are a large number of studies. Overall, the forest plot performed better in this experiment compared to the static version tested in Fitzgerald & Tipton,^12^ which suggests that providing actual study weight via hover text—as opposed to *only* encoding it as square size—may help improve users’ interpretation of forest plots.

**Table 4:** Tukey’s pairwise comparisons

Contrast	Difference Estimate	Lower CI	Upper CI	Adjusted p-value
MARCv2-BP	1.061	0.755	1.367	<0.001
MARCv2-FP	0.367	0.064	0.670	0.010
MARCv2-MARCv1	0.304	0.004	0.605	0.045
MARCv1-BP	0.757	0.452	1.062	<0.001
MARCv1-FP	0.063	−0.24	0.365	0.951
FP-BP	0.694	0.387	1.002	<0.001

***Research Question 3:** Do the visualizations perform consistently for all levels of k (# of studies)?*

**Figure 7:**
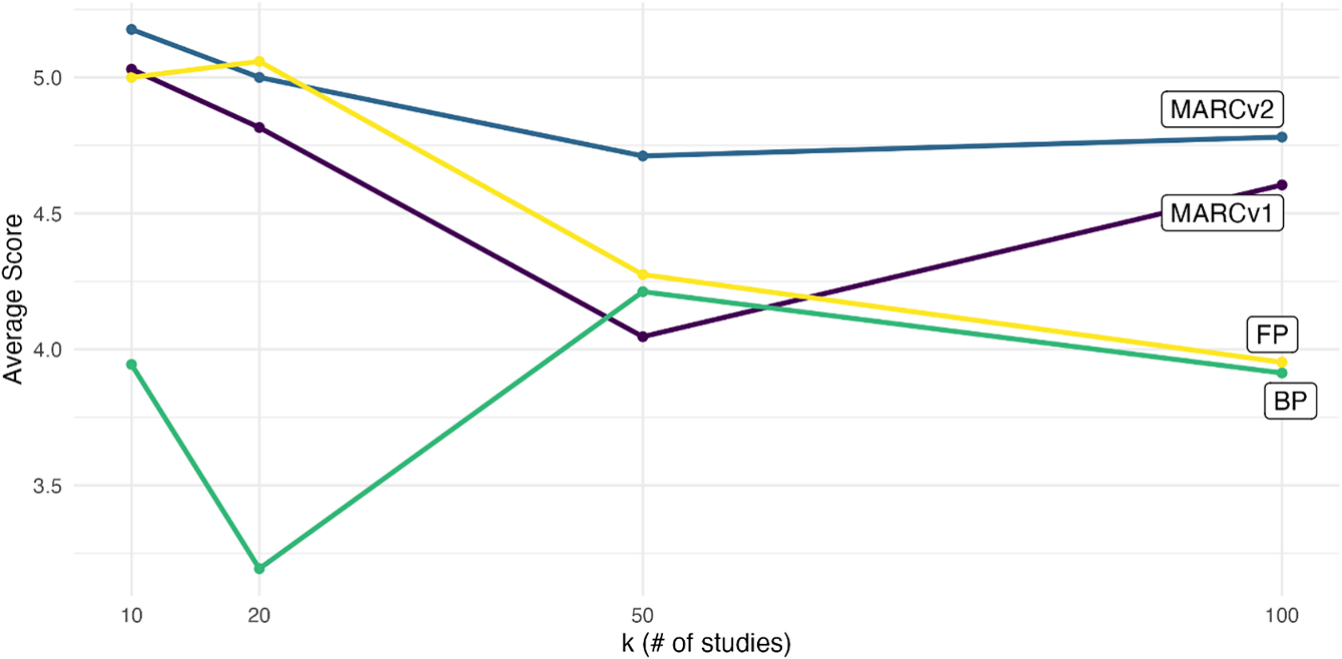
Average participant scores by number of studies and visualization type.

The ANOVA results for research question 3 are provided in Table 5, which indicates that the number of studies is not significant as a main effect, but the interaction between visualization type and number of studies is significant. The interaction plot in Figure 7 provides further insight; notably, MARCv2 performs relatively consistently for all values of *k*, whereas MARCv1, and especially the forest plot, seems to perform poorer with larger *k.* This suggests that MARCv2 may be less susceptible to getting visually cluttered in ways that can complicate the interpretation of the evidence for large meta-analyses. Note that the unusually low result for bar plots when *k* = 20 is likely because the *k* = 20 dataset was the only dataset where the study with the largest student sample size was not the study with the smallest standard error, which as discussed above, is particularly problematic for the bar plot. Therefore, this feature of the underlying data is more likely to be the cause of the dramatic dip seen in Figure 7, rather than being a true pattern pertaining to *k*. Indeed, when we run a robustness check by removing the observations for the bar plot with *k* = 20, the ANOVA results in a p-value of 0.042 for the VizType:K interaction term, which is no longer significant at the pre-specified level of 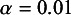 for this analysis.

**Table 5:** ANOVA results (RQ3)

Source of variation	Sum of squares	Degrees of freedom	*F*-statistic	*p*-Value
VizType	16.95	3	5.348	0.001
K	3.502	3	1.105	0.347
VizType:K	49.385	9	5.194	<0.001
PersonID	1357.11	157	8.182	<0.001
Intercept	145.161	1	137.401	<0.001
Residuals	460.623	436

***Research Question 4:** Does the length of time spent answering questions vary by visualization type? Does this relationship depend on k?*

To explore research question 4, we fit a two-level linear mixed effects model with repeated measurements (4) nested within people. Page duration (the amount of time in seconds spent answering questions for a particular visualization) was the outcome of interest and visualization type, number of studies (centered), and their interactions were included as Level 1 predictors (see the Appendix for details). Table 6 provides the model results.

**Table 6:** Model results (RQ4)

	Estimate	Standard error	*t*-Statistic	Lower CI (99%)	Upper CI (99%)
(Intercept)	199.264	10.813	18.428	171.507	227.02
MARCv1	6.077	13.854	0.439	−29.489	41.640
BP	33.961	14.071	2.414	−2.162	70.079
FP	36.382	13.954	2.607	0.581	72.243
k_centered	23.287	9.505	2.45	−1.17	47.757
MARCv1:k_centered	2.258	12.822	0.176	−30.709	35.156
BP:k_centered	−2.097	13.213	−0.159	−36.131	31.902
FP:k_centered	−2.812	13.225	−0.213	−36.781	31.129

**Figure 8:**
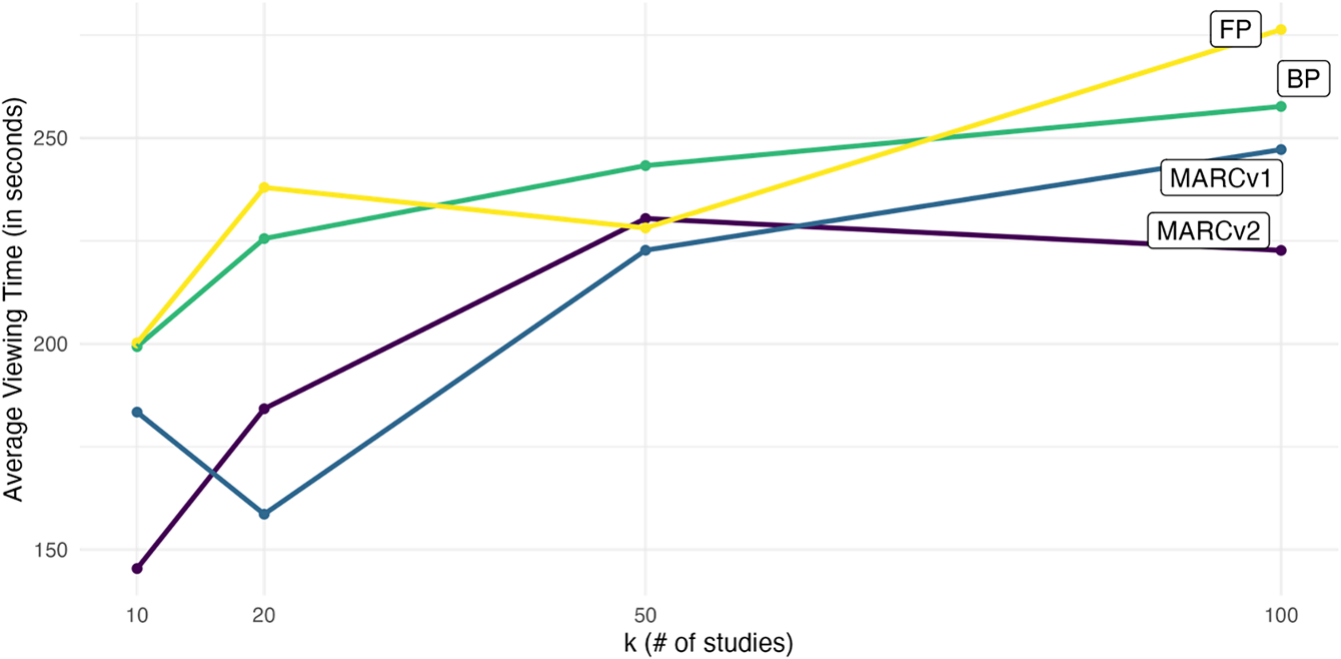
Average viewing time by number of studies and visualization type.

MARCv2 was used as the reference category, so the intercept represents the average viewing time for MARCv2, for an average number of studies. The positive slope estimates for MARCv1, BP, and FP indicate that the viewing time was shortest for MARCv2, on average. The only difference that was significant at the pre-specified 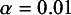 was the difference between forest plots and MARCv2, with participants taking on average 36 seconds longer to answer questions when viewing forest plots compared to MARCv2. Although the number of studies was not significant as a predictor, Figure 8 indicates that in general participants tended to take longer to interpret the evidence when there were more studies, across all visualization types.

## 6 Discussion and conclusion

Meta-analyses are increasingly important not only to researchers but also to the broader public faced with making decisions regarding policies and practices in education, medicine, criminology, and a variety of other fields. Although existing visualizations such as the forest plot may suffice for communicating meta-analytic data among researchers, careful attention must be paid to the curse of expertise and the ways in which a broader audience may not interpret statistical evidence as intended. We have found evidence that when data visualization and cognitive science best practices are intentionally incorporated into the design of a visualization (as in the MARC plot), we can substantially improve users’ interpretation of meta-analytic evidence.

The present findings extend results from Fitzgerald and Tipton^12^ to demonstrate that the MARC plot remains a more effective alternative to forest plots and bar plots for communicating to non-researcher audiences, even in the case of moderate and large meta-analyses. Importantly, however, in the case of larger meta-analyses, the adjusted MARC plot (MARCv2) should be used in favor of the original MARC design to avoid clutter and maintain the advantage of the *y*-axis positional encoding of meta-analytic weight. Code for generating both static and interactive versions of the MARC plot is available in the R package MARCviz, available via GitHub. See the Appendix for details.

While we did not study static versus interactive versions of the visualizations directly, we will provide the reader with a few considerations about this practical choice. For all visualization types studied (bar, forest, MARC plot), there is textual information that typically accompanies each effect size (e.g., study id label, sample size, weight), and it becomes infeasible to maintain this same textual information in a static visualization when there are a moderate or large number of effect sizes. So, the analyst is tasked with either removing the textual information or moving it into an interactive feature that becomes visible by some user action (e.g. hovering, clicking). Interactivity is a natural choice for providing rich information without visual clutter, and we did find some preliminary indication that interactive forest plots may do better than static ones, but the (interactive) MARC plot still outperforms the (interactive) forest plot. We recommend using interactivity when the communication medium allows (e.g., web-based dissemination), but of course there will likely always remain a need for static versions for non-web-based dissemination (e.g., pdf documents). While we have not empirically evaluated the static MARC plot for large meta-analyses (i.e., with textual information of effect sizes removed), we still expect it to be a better alternative to a static forest plot or bar plot in this scenario, for all of the design reasons discussed in this paper. One advantage of the MARC plot, regardless of interactivity, is that the size of the plot does not necessarily have to increase to accommodate more effect sizes. In a forest plot and bar plot, each new effect size adds another integer to the limits of the discrete *y*-axis, so to accommodate more effect sizes, the height of the plot must increase, or the space between the effect size/confidence interval bars must shrink, which can quickly become unreadable. This is why in practice researchers often avoid forest plots for large meta-analyses and may resort to tabular display only. Since the MARC plot is a scatterplot with a continuous *y*-axis, it does not suffer from this same limitation as the number of effect sizes increases. Larger meta-analyses will become increasingly common as the body of evidence continues to grow in every field, and MARC plots are a promising visualization to meet this demand. We similarly expect interactive displays to become increasingly important as the quantity and complexity of evidence increases across scientific fields, so we recommend further work to directly study interactivity and users’ engagement with it in order to establish best practices for interactivity in the dissemination of meta-analytic results.

Our present study has a few important limitations that point to other areas for further research. We only examined the case where there is one effect size per study. The MARC plot can easily be extended to handle the case of multiple effects per study by simply using an appropriate model (e.g. Robust Variance Estimation) for calculating the summary effect and its uncertainty. However, future empirical work is needed to evaluate best practices for visually communicating the grouping of effect sizes (e.g. by study ID or other characteristic) and to investigate if user interpretation of the evidence differs depending on how this grouping is made visually salient. Second, like forest plots, the MARC plot only depicts statistical uncertainty, but there are many other factors that contribute to the perceived trustworthiness and relevance of evidence by decision-makers (e.g. external validity, similarity to their context, intervention, and implementation details). Future empirical work is needed to understand how decision-makers holistically evaluate a body of evidence, how best to communicate non-statistical components needed for decision-making, and how best to measure decision-makers’ interpretation of and trust in more holistic information displays. Finally, participants in the present study were crowd workers on Prolific, and while this is common practice in data visualization and human-computer interaction research, further studies that recruit relevant decision-makers are necessary to continue establishing best practices for evidence communication.

The meta-analysis research community plays a crucial role in providing policymakers and practitioners with robust evidence to inform decision-making across many policy domains. Therefore, it is imperative that this evidence-focused community pays careful attention to and makes continued investments in the further development of best practices for not just meta-analysis but also evidence communication. Beyond the results of the particular experiment and recommendations presented here, we hope this study can serve as an example of the type of research that can be conducted to further establish best practices for evidence communication.

## Data Availability

This research was pre-registered with the Open Science Foundation, and all pre-registration materials, data, and code can be found in the OSF repository: https://osf.io/49ck7/.

## A Appendix

### A.1 Analysis plan

The following details the analysis plan for each research question, all of which were pre-registered with the Open Science Foundation, including multiple comparison adjustments.

*RQ1: Are users able to accurately interpret meta-analytic visualizations for meta-analyses with a moderate to large number of studies?*

For RQ1, we descriptively consider the proportion of respondents that are able to answer each Questions 1–7 correctly for each of the four visualizations. We also visually explore patterns in these proportions across the different levels of *k.* We did not conduct hypothesis tests for this research question.

*RQ2: Which type of visualization leads to the most accurate understanding among users?*

For RQ2, we define the following ANOVA model:

where  is the score for the individual  on visualization  , and  and  are the main effects of Factors A and B,  . The model also includes the two-way interaction  , the overall mean  , a blocking factor for individuals  , and the individual error term  . We test the six pairwise comparisons between the 4 levels of factor A using Tukey’s test of pairwise comparisons to control the “family-wise” Type I error rate.

*RQ3: Do the visualizations perform consistently for all levels of k (# of studies)?*

To investigate this question, we will test whether  and  are significant in the ANOVA model above. We also explore this question visually via an interaction plot.

*RQ4: Does the length of time spent answering questions vary by visualization type? Does this relationship depend on k?*

Here we fit a two-level linear mixed-effects model to account for repeated measurements (4) nested within people. We use a random intercept but fixed slopes and include visualization type (3 dummy variables  ), number of studies (*K*), and the *visualization***K* interactions in the Level 1 model. We use the better of the two MARC plots (MARCv2) as the reference category for visualization type and center *k* to aid in the interpretation of coefficients.

Level 1:

where

Level 2:

Note, that Level 1 represents the repeated measures (*j =* 1, 2, 3, 4) nested within individuals (Level 2, *i* = 1, 2, 3, …, *n*). The parameters γ_10_, γ_20_, and γ_30_ are of particular interest, as they represent the difference in average viewing time for each visualization compared to the reference visualization, for an average number of studies. We conduct *t*-tests on these three parameters. For example,  vs.  . Between RQ3 and RQ4 there are five hypothesis tests, so each one is tested at an  level.

### A.2 Online supplementary material

R code for creating static and interactive versions of MARCv2 can be found in the R package MARCviz, available via GitHub: https://github.com/kgfitzgerald/MARCviz.

The following supplementary materials can be found in the online OSF repository (https://osf.io/49ck7/):

- All pre-registration materials (preregistration.pdf)
- Online Appendices (Online_Appendices.pdf)
  - Online Appendix A: Experimental design details
  - Online Appendix B: Power Analysis
  - Online Appendix C: Generating meta-analytic data
  - Online Appendix D: Missing data
- All original, cleaned, and generated data
- R code for generating meta-analytic data (generate_MA_data.R)
- R code for creating experimental visualizations (viz_MARC.R, viz_adj_MARC.R, viz_barplot.R, viz_classicforest_kf.R)
- HTML displaying 16 experimental visualizations (visualizations.html)
- R code for cleaning and analysis to produce tables and figures in the manuscript (survey_data_cleaning.qmd, analysis_code.qmd)
- Qualtrics_Questionnaire.pdf
